# Phosphorylation of the mitochondrial autophagy receptor Nix enhances its interaction with LC3 proteins

**DOI:** 10.1038/s41598-017-01258-6

**Published:** 2017-04-25

**Authors:** Vladimir V. Rogov, Hironori Suzuki, Mija Marinković, Verena Lang, Ryuichi Kato, Masato Kawasaki, Maja Buljubašić, Matilda Šprung, Natalia Rogova, Soichi Wakatsuki, Anne Hamacher-Brady, Volker Dötsch, Ivan Dikic, Nathan R. Brady, Ivana Novak

**Affiliations:** 1 Institute of Biophysical Chemistry and Center for Biomolecular Magnetic Resonance, Goethe University, D-60438 Frankfurt, Germany; 2 Biomolecular Interaction Centre, School of Biological Sciences, University of Canterbury, Christchurch, 8020 New Zealand; 3 Structural Biology Research Center, Photon Factory, Institute of Materials Structure Science, High Energy Accelerator Research Organization (KEK), Tsukuba, Ibaraki 305-0801 Japan; 4 School of Medicine, University of Split, Šoltanska 2, HR-21000 Split, Croatia; 5 Lysosomal Systems Biology, German Cancer Research Center (DKFZ), Bioquant, Im Neuenheimer Feld 267, D-69120 Heidelberg, Germany; 6 Faculty of Science, University of Split, Ruđera Boškovića 33, HR-21000 Split, Croatia; 7 Structural Biology (School of Medicine), Beckman Center B105, 279 Campus Drive, Stanford, CA 94305-5126 USA; 8 W. Harry Feinstone Department of Molecular Microbiology & Immunology, Johns Hopkins University Bloomberg School of Public Health, Baltimore, MD USA; 9 Institute of Biochemistry II, Goethe University Medical School and Buchmann Institute for Molecular Life Sciences, D-60590 Frankfurt, Germany; 10 Systems Biology of Cell Death Mechanisms, German Cancer Research Center (DKFZ), Heidelberg University Hospital; Bioquant, Im Neuenheimer Feld 267, D-69120 Heidelberg, Germany; 11 Department of Surgery, Heidelberg University Hospital; Bioquant, Im Neuenheimer Feld 267, D-69120 Heidelberg, Germany

## Abstract

The mitophagy receptor Nix interacts with LC3/GABARAP proteins, targeting mitochondria into autophagosomes for degradation. Here we present evidence for phosphorylation-driven regulation of the Nix:LC3B interaction. Isothermal titration calorimetry and NMR indicate a ~100 fold enhanced affinity of the serine 34/35-phosphorylated Nix LC3-interacting region (LIR) to LC3B and formation of a very rigid complex compared to the non-phosphorylated sequence. Moreover, the crystal structure of LC3B in complex with the Nix LIR peptide containing glutamic acids as phosphomimetic residues and NMR experiments revealed that LIR phosphorylation stabilizes the Nix:LC3B complex via formation of two additional hydrogen bonds between phosphorylated serines of Nix LIR and Arg11, Lys49 and Lys51 in LC3B. Substitution of Lys51 to Ala in LC3B abrogates binding of a phosphomimetic Nix mutant. Functionally, serine 34/35 phosphorylation enhances autophagosome recruitment to mitochondria in HeLa cells. Together, this study provides cellular, biochemical and biophysical evidence that phosphorylation of the LIR domain of Nix enhances mitophagy receptor engagement.

## Introduction

Autophagy, a highly conserved cellular pathway responsible for recycling and degradation of cellular components and organelles, is essential for differentiation, development and homeostasis^1^. Abrogation of the autophagic pathway can contribute to a number of diseases, including cancer, neurodegeneration, infections and inflammation^2^. The key event of autophagy is the formation of autophagosomes, which are double membrane vesicles that occlude either non-selective or highly selective cargo that undergoes degradation after fusion with lysosomes. Bulk autophagy is induced by starvation and provides rapid supply of nutrients to starving cells. In contrast, selective autophagy is a precisely regulated process aimed to remove unwanted components (cargo) like protein aggregates, damaged organelles, etc, from the cytosol. It is based on specific cargo recognition by direct interaction of cargo receptors with members of the Atg8 protein family^3–9^. In humans, the Atg8 family comprises six proteins grouped in two sub-families - Microtubule-associated proteins 1 light chain 3 (LC3A, LC3B, LC3C), and γ-aminobutyric acid receptor-associated proteins (GABARAP, GABARAPL1 and GABARAPL2). All receptors described to date interact with these Atg8-proteins through a conserved tetrapeptide motif called LIR motif (LC3 Interacting Region). LIR motifs contain the core sequence ΘxxΓ, where Θ is an aromatic residue, Γ is a large hydrophobic residue, and x - any amino acid^10,11^. The essential residues of the LIR motif interact with two hydrophobic pockets on the surface of the Atg8 proteins to stabilize an intermolecular β-sheet formed by the LIR motif and β-strand 2 within the Atg8 proteins^12^. Frequent stretches of negatively charged residues occur directly prior to the LIR core, stabilizing the complex via interactions to positively charged residues in Atg8 proteins.

Of special interest is the selective autophagic removal of mitochondria, called mitophagy, that is mediated by Nix and Bnip3 proteins and occurs in cells to remove either damaged or superfluous mitochondria, thereby maintaining a healthy cellular environment^13,14^. The integral outer mitochondrial membrane protein Nix has been found to be essential for mitochondrial clearance during terminal differentiation of reticulocytes^15,16^. With a LIR motif on its N-terminal part exposed to the cytoplasm, Nix is a typical autophagy receptor that is able to bring mitochondria to autophagosomes through direct interaction with Atg8 family proteins^17^. The same behavior is common to its homolog Bnip3, which also serves as a mitochondrial autophagy receptor^14,18^. Recent data on Optineurin (Optn), an autophagy receptor for selective recognition and removal of intracellular bacteria (*Salmonella*) and protein aggregates, revealed that phosphorylation of serine residues juxtaposed to the LIR sequence is a major contributor to the specific interaction with Atg8 proteins^5^. Being phosphorylated on these serine residues, Optn binds stronger to LC3 proteins and therefore enforces xenophagy of *Salmonella*. Likewise, the phosphorylation state of the Bnip3 LIR determines receptor activity and induction of pro-survival mitophagy^14^. However, the detailed molecular mechanism of this signaling is unknown. Considering the evolutionary connection of xenophagy and mitophagy, and the similarity of Nix to the mitochondrial autophagy receptor Bnip3, we here studied the phosphorylation of serine residues (Ser34 and Ser35) adjacent to the LIR in Nix and the influence of this phosphorylation on the interaction of Nix with human Atg8 proteins. Our cellular, biochemical and biophysical analyses, including a crystal structure of the LC3B:Nix-LIR complex, revealed that the LIR phosphorylation status is essential for the fine-tuning of the LC3B:Nix interaction and crucial for the initiation of Nix-mediated mitophagy.

## Results

### Ser34 and Ser35 juxtaposed to the Nix LIR contribute to the interaction with LC3A/B through their phosphorylation

**Figure 1:**
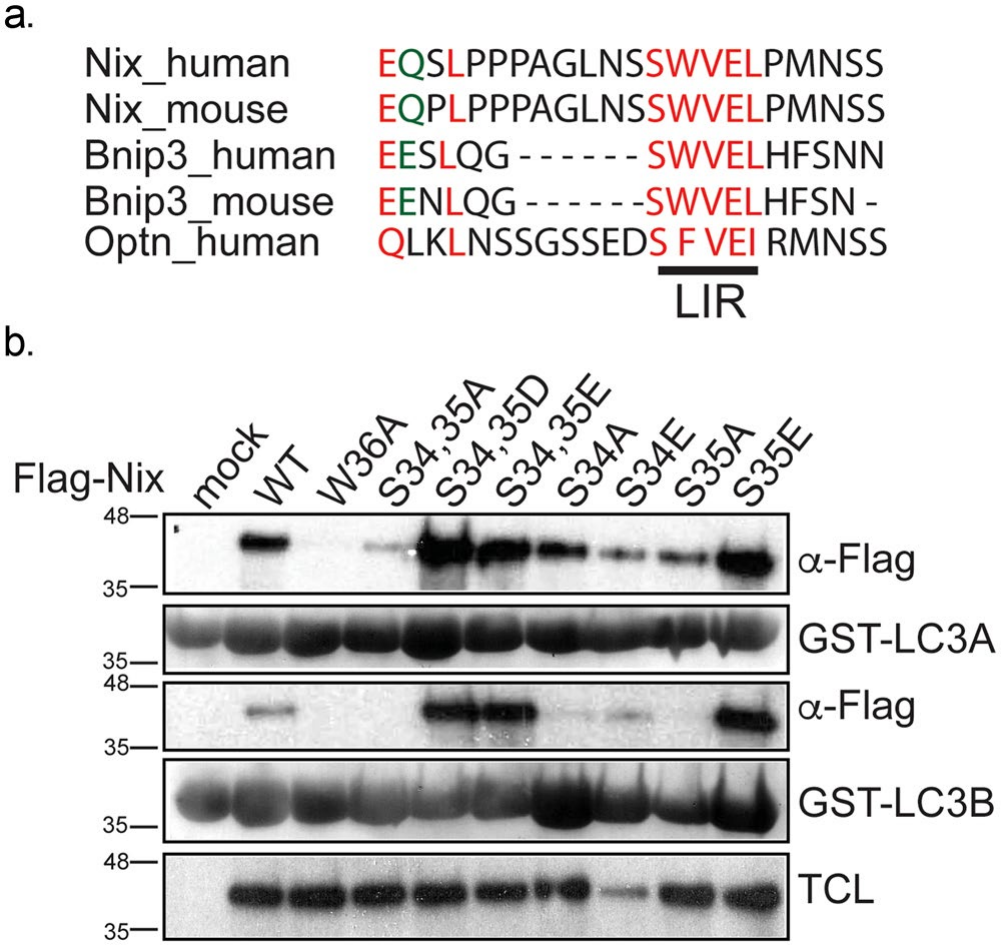
Phosphorylation of serine residues juxtaposed to LIR of Nix enhances interaction of Nix to LC3 protein family. (**a**) Alignment of LIR motif of Nix, Bnip3 and Optineurin. (**b**) GST pull-down of GST-LC3A and GST-LC3B and Nix wt and Nix W36A, double Nix S34,35A, Nix S34,35D, Nix S34,35E mutants and single Nix S34A, Nix S34E, Nix S35A, Nix S35E mutants. TCL corresponds to total cell lysate.

Interaction between autophagy receptors and ATG8/LC3/GABARAP proteins through specific LIR motifs has been shown to be important for proper activation of selective autophagy. Recently, it was determined that phosphorylation of serine residues juxtaposed to the LIR motif is responsible for the activation of receptor functions for Optn and Bnip3^5,14^. Comparative analysis of the mitophagy receptor Nix and its homolog Bnip3 revealed conserved serine residues N-terminal to the WVEL LIR sequence (Fig. 1a). Therefore, we mutated Ser34 and Ser35 in Nix to alanine, glutamic acid or aspartic acid residues, as well as Trp36 to alanine as a negative, binding-incompetent control^17^, and performed GST pull-down assays using GST-fused LC3A and LC3B. Previously we have shown that LC3B is the weakest Nix interacting partner^17^. Serine-to-alanine mutations, caused a complete loss of interaction with both LC3A and LC3B even with the non-mutated core LIR motif (Fig. 1b). This suggested that serine residues adjacent to the Nix LIR are as important for interaction with LC3 proteins as the core LIR itself. Conversely, phosphomimetic mutations of Ser34 and Ser35 to either glutamic or aspartic acid increased the affinity of their interaction with LC3A. Moreover, we observed that the affinity of the phosphomimetic Nix mutants to LC3B significantly increased as well (Fig. 1b). This is remarkable as the interaction of non-phosphorylated Nix with LC3B is considerably weaker than with LC3A (Fig. 1b)^17^. We also analyzed the interaction of the single Ser34 and Ser35 phosphomimetic mutants of Nix with GST-LC3A and GST-LC3B and could conclude that both S34E and S35E Nix mutants bind LC3A and LC3B stronger than wild type Nix but weaker than the double serine phosphomimetic S34,35D Nix mutant (Fig. 1b).

### Affinity and driving forces of interactions between Ser34, Ser35-phosphorylated Nix and LC3 proteins

**Figure 2:**
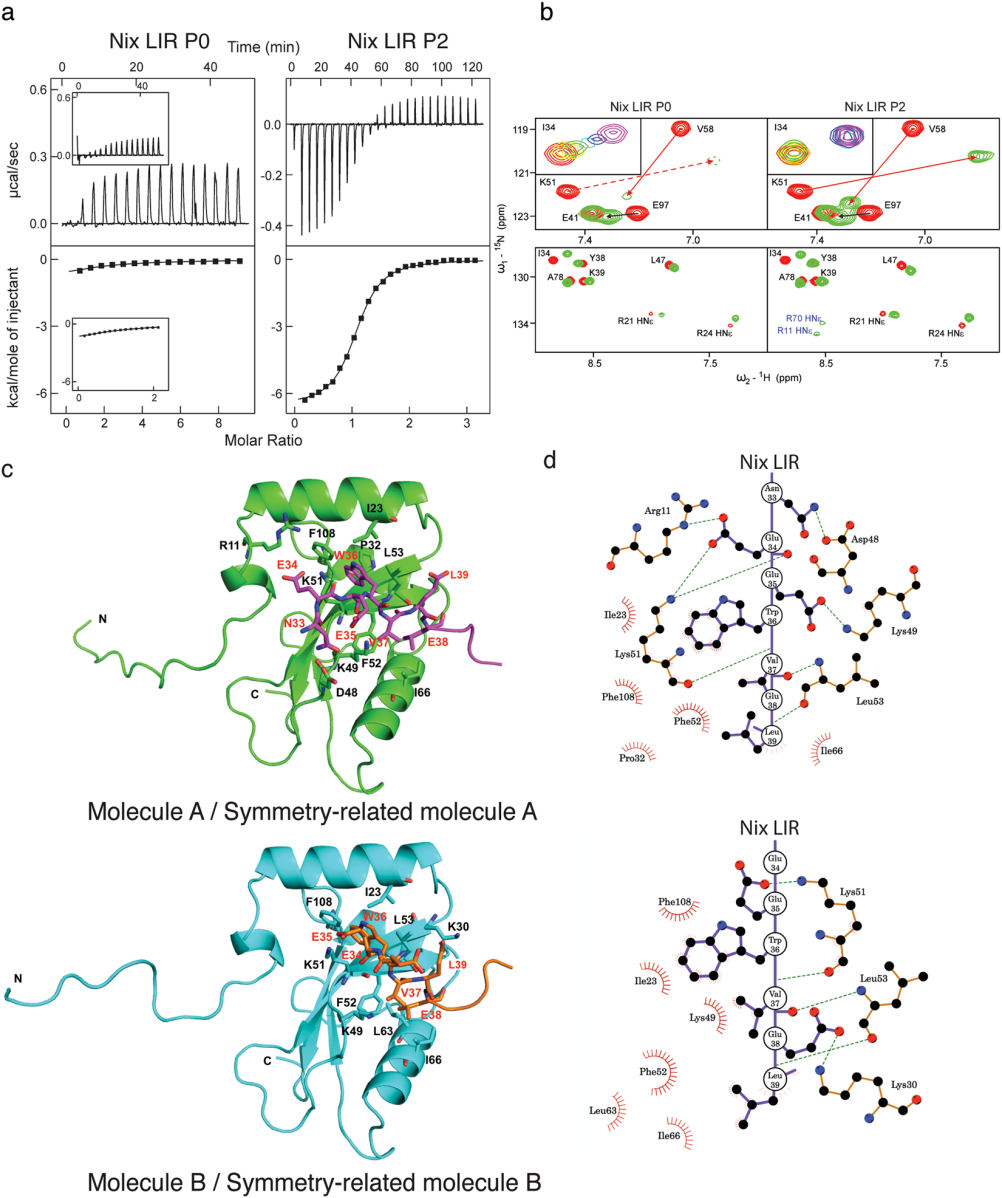
Influence of Nix phosphorylation on its interaction with LC3B studied by ITC, NMR and crystallography. (**a**) ITC profiles for the titration of Nix LIR P0 (left) and Nix LIR P2 (right) peptides into LC3B are shown at the main plots in the same scales of energy. For comparison, ITC data from Novak *et al*.^17^, representing LC3B titration with P0, are shown in the small windows within corresponding plot. In each plot the top diagram displays the raw measurements and the bottom diagram shows the integrated heat per titration step. (**b**) Upper plots: Representative sections (“fingerprint area”) of ^15^N-labeled LC3B [^15^N,^1^H]-TROSY HSQC spectra in the free LC3B state (red contours) and in complex with Nix LIR P0 and P2 peptides (green contours). Arrows show direction of CSP for selected residues. Dashed lines represent CSP of the K51 HN resonance, which cannot be visualized at this experimental series. Small windows within the plots represent exchange modes for the characteristic LC3B residues Ile34 upon P0 and P2 peptides titrations (rainbow color-code). Low plots: Representative spectral section showing arginine HN^ε^ side-chain resonances. They are aliased in the ω_1_ dimension and appear at a ^15^N chemical shift 50 ppm downfield from their true position. Tentative assignment for the HN^ε^ side-chain resonances appeared upon P2 titration is given in blue. (**c**) Structure of Nix/LC3B complex. Structures of Nix-LIR^S34,35E^-LC3B^2–119^ molecules A and B are represented by ribbon diagrams and colored in green (molecule A) and cyan (molecule B). Nix LIR portions in symmetry-related molecules (molecule A, magenta; molecule B, orange) and its interacting residues are shown in stick representation. Oxygen, nitrogen and sulfur atoms are shown in red, blue and yellow, respectively. Schematic representations showing the interaction between LC3B and Nix LIR^S34,35E^ on level of residues were generated by LIGPLOT^35^.

To characterize these interactions we used isothermal titration calorimetry and NMR titration experiments of unphosphorylated (AGLNSSWVELPMNSSNG, designated as P0) and phosphorylated (AGLNpSpSWVELPMNSSNG, designated as P2) Nix LIR peptides with LC3B. We have selected LC3B for further analysis as the weakest Nix interactor^17^, with potentially strongest enhancing of its affinity to Nix-LIR by phosphorylation. Additionally, LC3B is most studied and most understood protein within the human Atg8-family proteins, therefore the molecular mechanisms of phosphorylation-induced enhance of Nix-LIR:LC3B interaction will be more clear to evaluate. The ITC results (Fig. 2a, Table 1) showed that phosphorylation of Nix on Ser34 and Ser35 enhances its affinity to LC3B ~100 fold (K_D_ of 99 µM for P0 (91 µM reported in ref.^17^, for comparison see Table 1) and K_D_ of 0.83 µM for P2). Phosphorylation of the two serine residues increased the favorable enthalpy of interaction ΔH by 2 kcal/mol (from −2.58 to −4.40 kcal/mol, Table 1), indicating that new hydrogen bonds were involved in stabilizing the resulting complex. The entropies were positive both for binding of P0 and P2, therefore further enhancing the binding affinity.

**Table 1:** Thermodynamic parameters of interaction between LC3B (wild type and mutants), and Nix and p62 LIRs.

	ΔH kcal mol^−1^	ΔS cal mol^−1^ K^−1^	−T*ΔS kcal mol^−1^	ΔG kcal mol^−1^	K_a_ *10^6^ M^−1^	K_d_ μM	N
Nix LIR P0							
WT*	−2.72 ± 0.07	+9.4	−2.80	−5.52	0.011 ± 0.01	91	1**
WT	−2.58 ± 0.13	+9.7	−2.88	−5.46	0.010 ± 0.001	99	1**
K49A	−5.41 ± 0.50	+4.7	−1.40	−6.81	0.097 ± 0.009	10.3	0.98 ± 0.08
Nix LIR P2							
WT*	−6.57 ± 0.04	+5.7	−1.71	−8.27	1.17 ± 0.05	0.85	1.06 ± 0.01
WT	−4.40 ± 0.01	+13.1	−3.90	−8.30	1.21 ± 0.06	0.83	1.08 ± 0.01
R11A	−3.00 ± 0.01	+16.3	−4.86	−7.86	0.593 ± 0.05	1.69	1.06 ± 0.01
K49A	−5.44 ± 0.01	+9.9	−2.96	−8.40	1.43 ± 0.07	0.70	1.02 ± 0.01
K51A	+0.74 ± 0.01	+23.4	−6.97	−6.23	0.039 ± 0.01	26	1**
p62							
WT*	−10.5 ± 0.13	−8.7	+2.59	−7.97	0.68 ± 0.04	1.47	0.99 ± 0.01
WT	−9.38 ± 0.17	−5.4	+1.60	−7.78	0.498 ± 0.03	2.01	1.04 ± 0.01
R11A	−8.22 ± 0.23	−3.0	+0.90	−7.32	0.233 ± 0.05	4.29	1.06 ± 0.02
K49A	−10.84 ± 0.01	−8.9	+2.65	−8.19	1.02 ± 0.05	0.98	0.99 ± 0.01
K51A	−4.70 ± 0.01	+5.0	−1.48	−6.18	0.034 ± 0.01	29	1**

*Previous set of conditions reported by Novak *et al*.^16^ −50 mM NaPi, 150 mM NaCl, pH = 7.5.

**N was fixed to 1.00 upon fitting.

NMR titration experiments (Fig. 2b) did not reveal significant differences in the chemical shifts perturbation (CSP) pattern between the P0 and P2 peptides, similar to the results obtained for unphosphorylated and phosphorylated Optn-LIRs^19^. However, the higher binding affinity of the P2 peptide resulted in a shift from a fast-to-intermediate exchange behavior for the P0 peptide to slow exchange for the P2 peptide. This difference in the exchange dynamics can best be seen by analysis of the main chain amide proton of Lys51 which is involved in the formation of the intermolecular β-sheet between LC3B and all LIR motifs characterized so far. The resonance of Lys51 residue could not be detected in titration experiments with the unphosphorylated P0 peptide even at a 1:9 (LC3B:P0) molar ratio. However, this resonance was visible during the P2 titration, according to the earlier mentioned slow exchange behavior. A quantitative evaluation of the NMR titration experiments yielded a K_D_ of ~100 µM (Fig. 2b, small windows for Ile34, Supplementary Fig. S1a) for the P0 peptide, consistent with the observed fast-to-intermediate exchange. A slow exchange behavior as observed for the P2 peptide is similar to titrations with the canonical p62-LIR peptide, where the two resonance positions of the free and the bound LC3B can be detected simultaneously (Supplementary Fig. S1b).

Recent studies on the serine-phosphorylated Optn LIR sequence indicated the involvement of arginine side-chains in the recognition of the phosphorylated serine residues. Usually, side-chain protons of arginine residues exchange too fast with the water to be detectable. However, during the titration with the phosphorylated Optn LIR peptide they became detectable, showing that they are protected from exchange with the water by the formation of intermolecular hydrogen bonds^5,19^. This assumption was later confirmed by the NMR and X-ray structure determination of the LC3B/Optn LIR complex^19^. We have observed a similar effect for the Nix LIR motif (Fig. 2b, lower panels) as resonances of two arginine side-chains of LC3B became visible during the titration. On the other hand, these resonances could not be observed in titration experiments with the non-phosphorylated P0 peptide, suggesting that these side-chains are involved in interactions with the phosphorylated serines. The two arginine side-chain resonances were tentatively assigned as HN^ε^ resonances of LC3B Arg11 and Arg70.

### The structure of the complex of the phosphomimetic Nix LIR domain and LC3B provides the molecular mechanism of phosphorylation-regulated mitophagy

To further characterize the structural effect of Nix phosphorylation on LC3B binding, we crystallized a chimeric construct containing a phosphomimetic (two Ser were substituted with Glu) Nix LIR motif (Nix residues 28–39) fused C-terminally to LC3B (residues 2–119), Nix-LIR^S34,35E^-LC3B^2–119^, and solved the complex structure (2.35 Å, PDB code 4WAA). Data collection and refinement statistics are summarized in Table 2. The asymmetric unit of the Nix-LIR^S34,35E^-LC3B^2–119^ complex contains two molecules (molecules A and B) and the Nix LIR portion in each molecule interacts with its symmetry-related molecule (molecule A and B bind to symmetry-related molecule A and B, respectively). The binding mode is similar to previously reported LIR/LC3 complex structures^10^. The hydrophobic interaction surfaces of LC3B consist of Ile23, Pro32, Lys49, Lys51, Phe52, Leu53, Ile66, and Phe108 in molecule A, and Ile23, Lys49, Lys51, Phe52, Leu53, Leu63, Leu66, and Phe108 in molecule B. These LC3B residues interact with Trp36, Val37, and Leu39 of the Nix LIR in both molecules (Fig. 2c,d). The electron density of the N-terminal Nix region (PPAGLNEE) is weak compared to other regions or invisible (Supplementary Fig. S2). Therefore, we could not build a model of the Pro28 to Leu32 region of Nix. However, electron density could be observed for the Nix amino acids Asn33, Glu34, and Glu35 which were included in the model (Supplementary Fig. S2).

**Table 2:** Data collection and refinement statistics.

Parameter	Nix LIR^S34,35E^-LC3B (PDB code 4WAA)
Data collection	*P*4_3_2_1_2
Space group	
Cell dimensions	
a, b, c (Å)	67.4, 67.4, 116.9
α, β, γ (°)	90.0, 90.0, 90.0
Resolution (Å)	44.15–2.35 (2.48–2.35)
*R*_merge_	0.157 (0.567)
*I*/*σI*	15.7 (5.4)
Completeness (%)	100.0 (100.0)
Redundancy	13.9 (14.3)
Refinement	
Resolution (Å)	44.15–2.35 (2.41–2.35)
Number of reflections	11252
*R*_work_/*R*_free_	0.199/0.273
Number of atoms	
Protein	2082
Water	112
*B* factors	
Protein	24.9
Water	24.5
Ramachandran plot	
favoured (%)	98.8
allowed (%)	1.2
outlier (%)	0
RMSD	
Bond length (Å)	0.013
Bond angles (°)	1.558

Values in parentheses are for the highest-resolution shell.

Our structure suggests a mechanism by which phosphorylation affects LIR binding. In molecule A, the side-chain of Glu34 in the Nix LIR sequence interacts with the nearby Arg10 and Lys51 side-chains of LC3B and this suggests the possibility that hydrogen bonds are formed between Glu34^O^ ^ε1^ and Arg10^Nε^ (3.06 Å), and Glu34^O^ ^ε2^ and Lys51^Nζ^ (2.41 Å) (Fig. 2c and Supplementary Fig. S2b). In addition, a reorientation of the Arg11 side-chain to an alternative rotamer would place this side-chain in close proximity to Glu34, and presumably phosphorylated Ser34. In our structure, it is >4 Å away from Glu34 (Supplementary Fig. S2b). Given the weak density, the B-factor of Glu34 is higher than that of the overall structure (60.5 and 24.6), suggesting that Glu34 might be flexible in solution. Therefore, in the LIR bound state, the Glu34 residue very likely exists close to Arg10, Arg11 and Lys51 of LC3B and interacts with these side-chains.

It was recently reported that the side-chain of Lys49 of LC3A regulates the binding of LIR peptides through a gating mechanism^12^. The structural comparison of free LC3A and its complex with the Atg13 LIR motif demonstrated that the side-chain of Lys49 undergoes a 6.7 Å movement in the LIR bound state, providing access to the hydrophobic pocket and thereby allowing Val445 of the Atg13 LIR motif to form hydrophobic interaction with Phe52 and Lys49 of LC3A^12^. The increased binding affinity in the LC3A-K49A mutant is due to removing this Lys49 gating mechanism, allowing a direct access to the hydrophobic pocket 2. Based on the crystal structure (Fig. 2c,d), we propose that Lys49 of LC3B shows a similar gating mechanism since the Lys49 side-chain in our structure also moves by approximately 6 Å relative to the free protein.

### Role of phosphosensing LC3B residues in recognition of Ser34, Ser35-phosphorylated Nix

**Figure 3:**
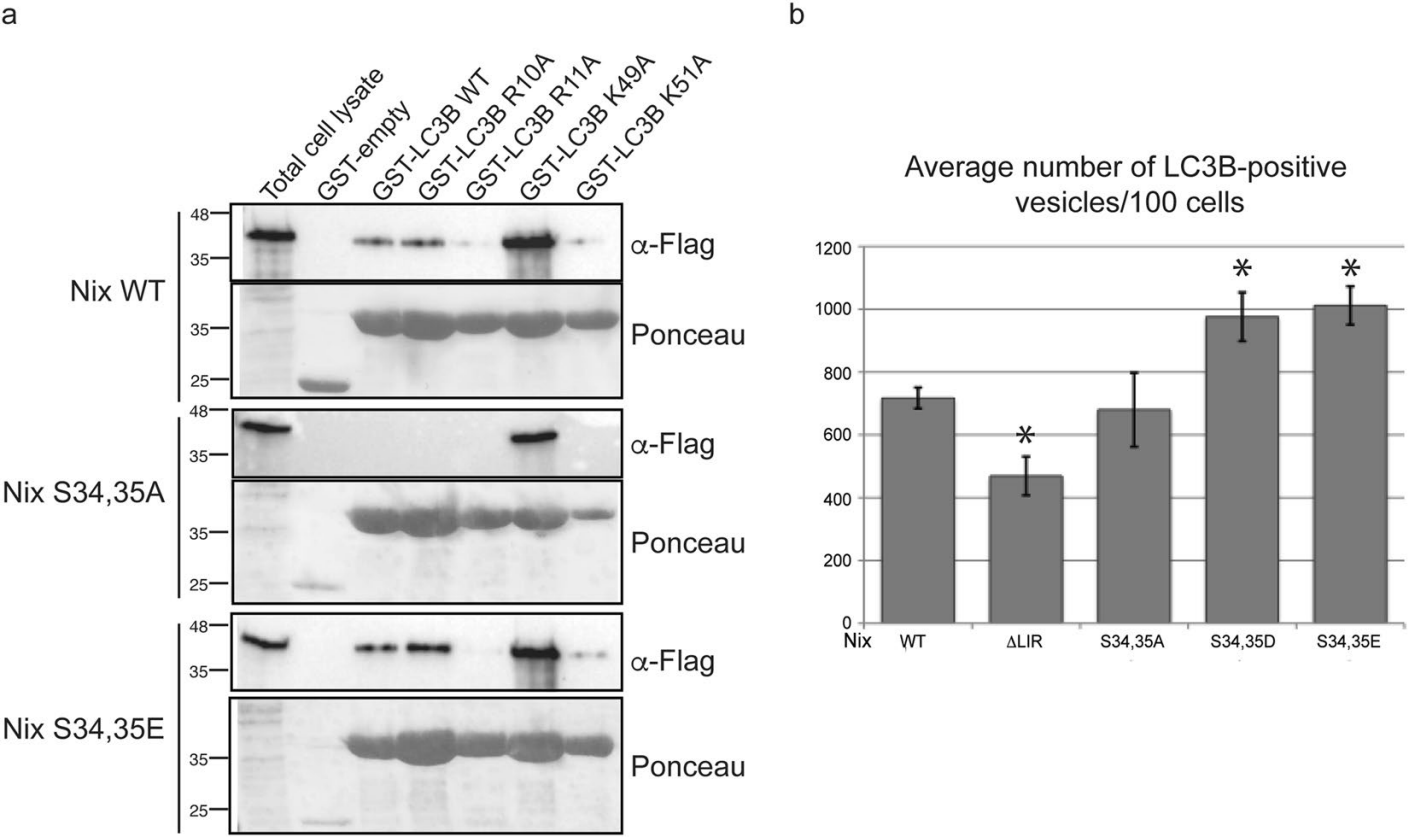
LC3B Arg10, Arg11 and Lys49 residues are important for interaction with phosphorylated Nix. (**a**) GST pull-downs with GST-LC3B wild type GST-LC3B, R10A, R11A, K49A and K51A were performed against Flag-Nix wild type, S34,35A and S34,35E. (**b**) Cells cotransfected with Nix wild type, ΔLIR, S34,35A, S34,35E or S34,35D and GFP-LC3B were screened using immunofluorescent microscopy. Three independent experiments were performed and average numbers of LC3B positive vesicles per 100 cells in each experiment were calculated. Error bars are standard deviation. *Significantly different from the control Nix wild type, *p* < 0.05.

To confirm our structural interpretation, we generated LC3B mutants of the amino acid residues involved in the observed interaction with Nix, i.e. GST-LC3B R10A, GST-LC3B R11A, GST-LC3B K49A and GST-LC3B K51A. We then performed GST pull-down assays with these GST-LC3B mutants and investigated the binding to wild type Nix and S34,35A and S34,35E mutants (Fig. 3a) in cell lysate. As expected, wild type LC3B weakly interacted with wild type Nix. Moreover, wild type LC3B did not interact with the Nix S34,35A mutant, but robustly bound phosphomimetic Nix S34,35E. Mutating positively charged amino acids R11A and K51A on LC3B interacting with the phosphomimetic residues on Nix strongly reduced interaction. Further, the LC3B K49A mutation strongly increased binding to all tested Nix proteins, including wild type and S34,35A mutants, consistent with the involvement of Lys49 in the gating of the hydrophobic pocket, as seen for LC3A^12^ (Supplementary Fig. S3). These results confirm the involvement and essential role of Arg11, Lys51 and Lys49 residues of LC3B in the interaction with Nix as shown in the presented crystal structure; and indicates that Arg10 is less important for binding of Ser34,35-phosphorylated Nix *in vivo* (despite our Nix-LIR:LC3B structure indicates higher importance of Arg10 and not Arg11).

To confirm our interaction data on a cellular level, we tested the recruitment of LC3B to mitochondria in HeLa cells overexpressing wild type Nix, ΔLIR, phosphomimetic mutants (S34,35D and S34,35E) or serine-to-alanine mutations to disable phosphorylation (S34,35A). First, we confirmed by immunofluorescence microscopy that the generated Nix mutants localized to Tom20-immunolabeled mitochondria (Supplementary Fig. S4). Next, transfected HeLa cells were treated with 10 µM CCCP for 2 hours to boost recruitment of the autophagy machinery and the number of LC3B-positive vesicles was evaluated as described in the Methods section. The phosphomimetic mutants (S34,35D and S34,35E) showed increased recruitment of LC3B-positive vesicles compared to wild type and S34,35A. This difference is further enhanced when S34,35D and S34,35E are compared to the ΔLIR mutant (Fig. 3b).

**Figure 4:**
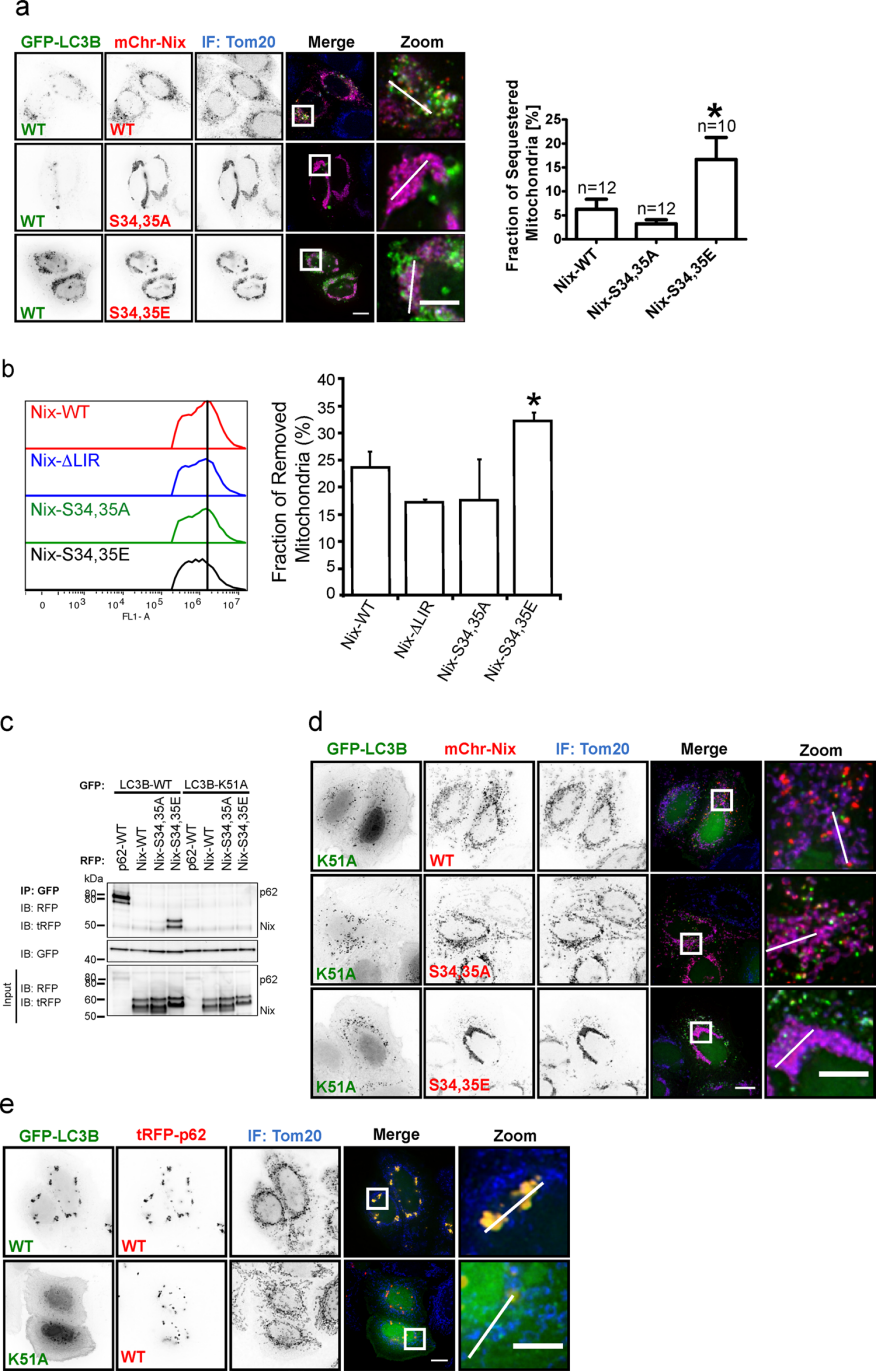
Phosphorylation of the Nix LIR influences the initation of mitophagy and Nix:LC3B interaction depends on LC3B Lys51. (**a**) Hela cells coexpressing GFP-LC3B wild type and Nix wild type or mutants, immunostained (IF) for mitochondrial marker Tom20. GFP-LC3B shows colocalization with RFP-Nix and enhanced colocalization with S34,35E mutant. RFP-Nix S34,35A does not colocalize with GFP-LC3B. Analysis of fraction of sequestered mitochondria is depicted on the graph. (**b**) Analysis of mitochondrial removal is determined by flow cytometry using GFP-Nix, ΔLIR, S34,35A and S34,35E mutants (**c**) Immunoprecipitation of GFP-LC3B wild type or GFP-LC3B K51A in cells co-expressing tRFP-p62 wild type, RFP-Nix wild type, S34,35A and S34,35E mutant. GFP-LC3B K51A fails to coimmunoprecipitate with p62, Nix wild type and mutants. (**d**) GFP-LC3B K51A does not colocalize with Nix wild type or mutants. (**e**) Hela cells coexpressing GFP-LC3B wild type or K51A mutant and RFP-p62, immunostained (IF) for Tom20. Scale bar 10 µm.

Using mutant and wild type GFP-tagged LC3B as well as RFP-tagged Nix and Bnip3, we investigated the cellular mitophagy response. Consistent with the above results, Nix S34,35E elicited an enhanced targeting of mitochondria to LC3B autophagosomes relative to wild type Nix. Importantly, Nix S34,35A resulted in a lack of such a recruitment (Fig. 4a, Supplementary Fig. S5b). In support of these imaging data, in immunoprecipitation experiments with GFP-LC3B, we detected no interaction with Nix wild type or Nix S34,35A, but strong interaction with Nix S34,35E (Fig. 4c).

Furthermore, we have performed flow cytometry experiments to evaluate the phosphomimetic effects of Nix on the mitochondrial loss during mitophagy. We have transfected HEK293 cells with GFP-Nix wild type, ΔLIR, S34,35A or S34,35E, respectively and treated for 24 hours with 10 μM CCCP. We have analyzed fluorescently labeled mitochondria and could show that the mitochondrial loss is more rapid in cells transfected with S34,35E mutant compared to wild type (presented as a fraction of removed mitochondria) (Fig. 4b). As a control we have used ΔLIR and S34,35A mutants where we have observed that mitochondrial degradation is slightly reduced compared to wild type (Fig. 4b). Further, as a control, we have analyzed the mitochondrial retention in the cells treated with 10 μM CCCP/100 nM Bafilomycin A to block the lysosomal degradation step (Supplementary Fig. S6b) and the slight accumulation of the mitochondria is seen in all mutants. All indicates that indeed, phosphorylation of S34,35 in Nix could increase mitophagy not only in initial stages of the process but also in respect of mitochondrial removal.

To evaluate the role of critical residues in LC3B that sense the presence of phosphorylated or negatively charged residues we performed co-localization and immunoprecipitation experiments with the K51A mutation in LC3B. Lys51 is a highly conserved residue in all Atg8-proteins ranging from yeast to human. Its aliphatic part contributes to the formation of the hydrophobic core 1, whereas its charged amino group forms hydrogen bonds and salt-bridges to negatively charged residues. In support of our findings the LC3B K51A mutant was unable to immunoprecipitate Nix S34,35E from cell lysates (Fig. 4c). The pivotal role of Lys51 as a Nix phosphosensing residue was further supported by loss of cellular colocalization of LC3B K51A labelled autophagosomes and Nix S34,35E decorated mitochondria (Fig. 4d, Supplementary Fig. S5b). Similarly, while wild type LC3B strongly bound Bnip3S17,24E, supporting the results published earlier^14^, this interaction was absent with the LC3B K51A mutant (Supplementary Fig. S7).

Immunoprecipitation experiments of the LC3B R11A mutant with wild type Nix or its phosphomimetic mutant resulted in similar effects (Supplementary Fig. S5a). Further, when cellular colocalization of LC3B K49A with Nix S34,35E was monitored, it was increased which is in agreement with its role as a gate keeper residue (Supplementary Fig. S3).

As a positive control, we used p62. The p62 LIR motif contains three negatively charged residues prior to the core LIR tetrapeptide (DDDWHEL) and the structure of the complex of p62 LIR and LC3B shows that the Lys51 side-chain forms hydrogen bonds to D339 of p62 LIR. Mutation of Lys51 strongly decreased the affinity to p62^20^. In our experiments, while p62 bound and localized with wild type LC3B, this interaction was lost with the LC3B K51A mutant (Fig. 4c,e, Supplementary Fig. S5b), showing that Lys51 is a general sensor of negative charges directly N-terminal to the LIR sequence. Similar to Nix, the LC3B R11A showed reduced co-localization to p62, while LC3B K49A increased it.

To further quantify the effect of these mutations we used ITC titration experiments (Supplementary Fig. S8 and Table 1). As expected the LC3B R11A mutation reduced the binding to both p62 and the Nix P2 peptides while for the LC3B K51A mutant no interaction with p62 and to P2 Nix LIR peptides could be detected. In contrast, the LC3B K49A mutant shows increased binding affinity to both peptides (K_D_ of ~100 μM for wild type LC3B and ~10 μM for LC3B K49A mutant); this effect is even more pronounced for the P0 Nix LIR peptide (Table 1). Interestingly, the same LC3B K49A mutation did not increase the affinity to the arbitrary LIR-similar peptide (SQQGGWGTVFSLESEE, residues 196–210 of the human protein BCL2L13 - analog of the yeast Atg32 protein). This LIR-like peptide possesses no negatively charged residues prior to the aromatic amino acid and its affinity to both wild type LC3B and the LC3B K49A mutant remained weak (Supplementary Fig. S8c, right plot), indicating a specific action of this mutation for the real LIRs.

Overall, our findings indicate that phosphorylation of the Nix LIR strongly enhances Nix interaction with Atg8 homolog proteins and increases recruitment of the autophagic machinery to the targeted mitochondria and thereby improves the efficiency of mitochondrial sequestration.

## Discussion

Nix is an atypical BH3-only protein able to induce a mitochondria-mediated cell death program^21^. Similar to Bnip3^22^, the pro-apoptotic activity of Nix depends on both its BH3 and transmembrane domains^21^. Nix is associated with activation of both apoptotic and necrotic cell death during cardiac hypertrophy^23,24^, and is a putative tumor suppressor^25^. In addition, during erythrocyte differentiation Nix drives LIR-mediated mitochondrial clearance^17^, in a manner independent of apoptosis signaling^16^.

Intriguingly, the LC3-binding activities of known mitochondrial autophagy receptors, Fundc1, Bnip3 and Nix, are all regulated via phosphorylation near their LIR motifs^14,26^. It was reported recently that Bnip3-induced mitophagy can pre-condition cells against mitochondrial apoptosis^14^, demonstrating that the LIR phosphorylation state dictates pro-death versus pro-survival function of Bnip3. Given the functional homology between BH3, transmembrane and LIR domains of Nix and Bnip3, it is likely that phosphorylation of the Nix LIR domain results in the same multi-functionality.

Bnip3 and Nix share a conserved core LIR motif preceded with Ser residue (Fig. 1a). Previously, through mutagenesis of Ser17 in Bnip3 to aspartic acid, it was shown that serine phosphorylation of Bnip3 is also required for LC3B and GATE16 binding^14^. However, the structural basis for this requirement remained unknown. In this work we describe a structurally-based mechanism of enhanced binding between LC3B and phosphorylated Nix proteins and demonstrate that Lys51 is crucial for both Nix and Bnip3-induced mitophagy (Figs 4 and S3). Interestingly, a Lys to Glu mutation at the equivalent position (K48) of yeast Atg8 does not perturb its interaction with the Atg19 LIR motif (which does not possess negatively charged residues prior to the Trp residue - ALTWEEL) and does not affect Atg8 autophagosomal localization. However, the K48E Atg8 mutant was defective in autophagic transport, indicating loss of the Atg8-LIR interaction with proteins downstream of Atg8 lipidation and membrane incorporation^27^.

We also observed that the LC3B K49A mutation resulted in increased affinity to both Nix (Fig. 3) and Bnip3 (Supplementary Figs S3 and S7). Lys49 is another highly conserved residue in Atg8-proteins, acting as a gatekeeper to regulate the interaction of large hydrophobic residue at LIR position +4 with the hydrophobic pocket 2^12^. Interaction of Lys49 with phosphorylated or negatively charges residues N-terminal to the core LIR motif remove the Lys49 side-chain from its gating position, thus allowing access to the hydrophobic pocket. Substitution of Lys49 to Ala inactivates this gating mechanism, therefore resulting in higher binding affinity to LIR sequences, as it was shown both by ITC and cellular colocalization experiments. Interestingly, the mutational analysis of Arg10 and Arg11 shows higher significance of Arg11 for the binding of phosphorylated Nix. In our X-ray structure, it is Arg10 side-chain participating in intermolecular hydrogen bond to the phosphomimicking Glu34. However, this area (first two α-helices) of all Atg8-family proteins is highly mobile and we could not exclude that in solution there are some conformational equilibria turning Arg11 to a more important sensor of negatively charged residues prior to aromatic (W/Y/F) residues in LIR-motifs as it was shown earlier^19^.

While both Ser34 and Ser35 of Nix are involved in binding to LC3B, Bnip3 contains only a single serine (Ser17) residue directly N-terminal to the core LIR sequence. Further work will be required to address whether different serine phosphorylation patterns regulate different binding affinities of Bnip3 and Nix with particular Atg8 family members^14,17^. S34,35 phosphorylation *in vivo* is rather a possibility for regulation of mitochondrial clearance; direct detection of Nix phosphorylation at residues Ser34 and Ser35 was not possible in our experiments. However, flow cytometry of cells expressing Nix wt, Nix S34,35A and S34,35E mutants, suggests that phosphorylation of these serines may be required for effective Nix-mediated mitophagy *in vivo*.

In conclusion, this work shows that phosphorylation of residues Ser34 and Ser35 in Nix enables Nix binding to human Atg8-family proteins and therefore initiates Nix mitophagy function. We present here the first atomic detailed structure of phosphorylation-based regulation of interactions between mitochondrial autophagy receptors and Atg8 homolog proteins.

## Material and Methods

### Plasmids

Plasmids used in the study were generated by site-directed mutagenesis by PCR to introduce desired mutations in the Nix constructs. The correctness of the DNA sequence was verified by sequencing. Plasmids used were the following: pEGFP-C1/Nix wt; pEGFP-C1/Nix W36A, pEGFP-C1/Nix S34,35A; pmCherry-C1/Nix S34,35A; pmCherry-C1/Nix S34,35E; pEGFP-C1/Nix S34,35D; pEGFP-C1/Nix S34,35E; pcDNA3.1/Flag-Nix wt; pcDNA3.1/Flag-Nix W36A, pcDNA3.1/Flag-Nix S34,35A; pcDNA3.1/Flag-Nix S34,35D; pcDNA3.1/Flag-Nix S34,35E; pcDNA3.1/Flag-Nix S34A; pcDNA3.1/Flag-Nix S34D; pcDNA3.1/Flag-Nix S34E; pcDNA3.1/Flag-Nix S35A; pcDNA3.1/Flag-Nix S35D; pcDNA3.1/Flag-Nix S35E; pEGFP-C1-LC3B, pEGFP-LC3B K49A, pEGFP-LC3B K51A, ptagRFP-p62, pGEX-4T1/LC3A; pGEX-4T1/LC3B; pGEX-4T1/LC3B R10A; pGEX-4T1/LC3B R11A; pGEX-4T1/LC3B K49A; pGEX-4T1/LC3B K51A; pmCherry-C1/Bnip3 wt; pmCherry-C1/Bnip3 S17,24A; pmCherry-C1/Bnip3 S17,24E; pET30Δ^28–39]^, ^S34,35E^-LC3B, pETm60_Ub3-LC3B.

### Antibodies and reagents

The following antibodies were used in the study: mouse monoclonal anti-Flag M2 (Sigma, 1:10,000); rabbit polyclonal anti-GFP (Clontech, 1:1000); mouse monoclonal anti-GFP (Roche, 1:1000), rat monoclonal anti-GFP (Chromotek, 1:1000), rat monoclonal anti-RFP (Chromotek, 1:1000), rabbit polyclonal anti-tRFP (Evrogen, 1:3000), rabbit polyclonal anti-Tom20 (Santa Cruz Biotech, 1:1000), mouse monoclonal anti-cytochrome c (Santa Cruz, 1:1000), mouse monoclonal anti-actin (Sigma 1:3000), rabbit polyclonal anti-LC3 (a kind gift from Z. Elazar), secondary HRP, FITC or Cy3 conjugated antibodies, goat anti-mouse and goat anti-rabbit IgGs were used for immunoblotting.

### Immunofluorescence microscopy

HeLa cells were seeded, grown on coverslips or 8-well microscopy slides (ibidi) and transfected with either wild type or mutant Nix or Bnip3 together with wild type or mutant LC3B constructs using JetPRIME transfection reagent according to manufacturer’s instructions. 24 hrs post transfection cells were fixed in 2% or 4% paraformaldehyde and permeabilized with 0,1% Triton X-100 solution. Cells were blocked in 5% BSA/PBS/0.1% Triton X-100 for 1 hr at room temperature or 4 °C overnight. Primary and secondary antibodies were diluted in blocking solution and washed in 0,1% Triton X-100/PBS. Coverslips were mounted in Prolong mounting media (Invitrogen). Cells in 8-well microscopy slides were placed in PBS following staining. Cells were imaged using a Zeiss AxioVision or DeltaVision RT microscope system (Applied Precision). Quantification of Nix-LC3B colocalization was performed as follows: for each Nix construct, LC3B positive dots (signals) were numbered in 100 cells. Only clear and well-defined LC3B signals are taken into consideration, while weak and oversized signals were excluded from the analysis.

### Quantifications of mitophagy from fluorescence microscopy data

HeLa cells were plated in 8-well microscopy µ-slides (ibidi) were transfected GFP-LC3 and RFP-Nix WT, S34,35A or S34,35E. From Z-stacks, single cells were cropped for analysis by ImageJ. Binary masks for each slice within ≥10 representative Z-stacks per condition of (i) Nix-labeled mitochondria and (ii) GFP-LC3 were generated by image segmentation. Slice-by-slice co-localization of mitochondrial and AV masks were calculated using the Boolean AND function. All slices for each binary stack were summed, and the ratio of area calculated from mitochondrialy-localized AV over total mitochondria content is reported as a cellular fraction.

### Preparation of GST-fusion proteins

GST fusions of various proteins (pGEX plasmids) were expressed in BL21 *E*. *coli*. Fusion protein expression was induced by adding 0.5 mM IPTG for 4 hrs. Bacteria were lysed in 20 mM Tris-HCl, pH 7.5, 10 mM EDTA, pH 8.0, 5 mM EGTA, 150 mM NaCl supplemented with 2 mg/ml lysozyme. GST fusion proteins were subsequently bound to pre-washed Glutathione-Sepharose 4B beads (GE Healthcare). After several washes, fusion protein-bound beads were used directly in binding assays.

### Preparation of 6xHis-fusion proteins

6xHis fusion of Nix LIR-LC3B protein (pET30Δexpressed in BL21(DE3) *E*. *coli*. The protein expression was induced by adding 0.3 mM IPTG for 16 hrs at 25 °C. Bacteria were lysed in 20 mM Tris-HCl, pH 9.0, 100 mM NaCl, 10 mM Imidazole pH 8.0 supplemented with 1% Triton X-100. The protein was subsequently bound to Ni-NTA (Invitrogen). After several washes, attached protein was cleaved with thrombin (GE healthcare) at 22 °C for 16 hrs. Eluted protein was further purified by gel-filtration (Superdex 200 10/300 GL, GE Healthcare).

### Preparation of LC3B protein for NMR and ITC experiments

For ITC and NMR studies, the non-labeled and ^13^C,^15^N-labelled LC3B protein was obtained using optimized Ub-fusion technology^28^ based on the protocols described elsewhere^10,19^. The 95% pure peptides representing Nix-LIR motif without and with phosphorylation of Ser34 and Ser35 (respectivelly, P0: AGLNSSWVELPMNSSNG, and P2: AGLNpSpSWVELPMNSSNG) were purchased from GenScript USA Inc (N.J., USA). Before the experiment, the proteins and peptides were equilibrated with a buffer containing 40 mM Na_2_HPO_4_, 80 mM NaCl at pH 7.0, and supplied with 5 mM protease inhibitors cocktail.

### GST pull-down assay

HEK293 or HeLa cells were transfected with expression constructs encoding the protein of interest using JetPRIME (Polyplus Transfection). 24 hrs post-transfection cells were lysed in 50 mM HEPES, pH 7.5, 150 mM NaCl, 1 mM EDTA pH 8.0, 1 mM EGTA, 10% glycerol, 1% Triton X-100, 25 mM NaF, 50 mM ZnCl_2_ and lysates were incubated for 4 hrs with immobilized GST-fusion proteins at 4 °C. Following 5 washes, beads with co-precipitated proteins were resuspended in 2x SDS-PAGE loading buffer, boiled and loaded onto 10% or 12% SDS-PAGE gels for analysis. All GST pull-down experiments were performed in triplicates.

### Co-Immunoprecipitation

Cells were scraped, centrifuged at 500 g for 5 min, and lysed in 150 µL CHAPS lysis buffer (2% CHAPS, 1% Triton X-100, 137 mM NaCl, 2 mM EDTA, 10% glycerol, and 20 mM Tris-HCl, pH 8.0) containing protease and phosphatase inhibitors. 5 µL of total lysate was kept as Input sample and 145 µL of cell lysate was incubated with 12.5 µL α-GFP (1:40, Roche)-coupled Dynabeads Protein G (purchased from Invitrogen) for 2 hrs at 4 °C. This step was followed by three washing steps using 0.02% Tween-20/PBS and immunoprecipitated proteins were eluted from Dynabeads by adding 4X LDS buffer (purchased from Invitrogen) and 10X sample reducing agent (purchased from Invitrogen) to the beads. Heating at 70 °C for 10 min will denaturized the proteins to analyze them by Western blotting.

### Flow cytometry

HEK293 cells were transfected with GFP expression constructs encoding the protein of interest using JetPRIME (Polyplus Transfection). 24 hrs post-transfection cells were treated with 10 µM CCCP for 24 hrs. After treatment, cells were washed with PBS, trypsinized with trypsin for 5 min and resuspended by gentle pipetting. Trypsin-mediated digestion was arrested by the addition of DMEM supplemented with 10% FBS and cells were centrifuged at 500 g for 5 min. To remove cell clumps, cells were resuspended in PBS and filtered through the 70 µm Cell Strainer (BD Falcon). Approximately 10^6^ cells were acquired for further flow cytometry analysis. Before fixation, cells were washed twice with FACS buffer (2% FBS, 0,02% NaN_3_ in PBS) and each time centrifuged at 500 g for 5 min. Cells were fixed with freshly prepared 4% paraformaldehyde for 10 min at room temperature, washed twice with FACS buffer and finally resuspended in of FACS buffer prior to analysis. 100000 events were collected on the BD Accuri 6 flow cytometer (Beckman Coulter) with PI collected in the FL3 channel and GFP in the FL1 channel. The acquired data were gated for singlets by generating FSC-A vs FSC-H plot after which dead cells were excluded and the mean fluorescence in FL1 channel was collected. The mean fluorescence of the CCCP and CCCP/Baf treated samples was compared with the untreated control and the results were expressed as a percentage of the retained mitochondria of at least two independent experiments. Results were analyzed using FlowLogic software.

### Isothermal Titration Calorimetry (ITC)

All titration experiments were performed at 25 °C using a VP-ITC microcalorimeter (MicroCal Inc., MA, USA). The ITC-data were analyzed with the ITC-Origin 7.0 software with a “one-site” binding model. The peptides at concentrations of 0.31 mM were titrated into 0.018 mM LC3B proteins in 25 steps. The proteins and peptides concentrations were calculated from the UV-absorption at 280 nm by Nanodrop spectrophotometer (Thermo Fisher Scientific, DE, USA).

### Nuclear Magnetic Resonance (NMR) spectroscopy

All structural NMR experiments were performed at 298 K on Bruker Avance spectrometers operating at proton frequencies of 600 MHz. Titration experiments were performed with a 0.15 mM ^13^C,^15^N-LC3B protein sample to which non-labeled synthetic Nix-LIR peptides in different phosphorylation states were added stepwise until 8 times excess of the non-phosphorylated P0 peptide, or 2 times excess of the phosphorylated P2. Backbone resonances for LC3B in complex with the P2 peptide were assigned using [^15^N-^1^H]-TROSY versions of 3D HNCACB experiment.

### X-ray crystallography

Initial crystallization screening was performed using kits from Hampton Research (Crystal Screen and Crystal Screen 2, PEG/Ion Screen 1 and 2, MembFac and Index), from Emerald BioStructures (Wizard I and II, Cryo I and II), and from Molecular Dimensions (Stura Foot print Screens) by sitting-drop vapor-diffusion method at 20 °C using an automated crystallization robot system^29^. The crystal of a chimeric construction containing a phosphomimetic (two Ser were substituted with Glu) Nix LIR motif (Nix residues 28–39) fused C-terminally to LC3B (residues 2–119), Nix-LIR^S34,35E^-LC3B^2–119^, was obtained using PEG/Ion Screen 2 condition No. 10 [8% Tacsimate, pH 4.0, 20% (w/v) PEG3350]. Diffraction data were collected at beamline AR-NW12A at Photon Factory (Tsukuba, Japan). The crystal was mounted in a nylon fiber loop and flash-cooled in a nitrogen-gas stream. The diffraction data were indexed and integrated with Mosflm^30^, and scaled with Scala^31^. The structure was solved by the molecular replacement with MOLREP^32^ using the structure of LC3B^2–119^ as a search model (PDB ID, 3VTU^19^). The model was refined with REFMAC5^33^. Manual adjustments of the structure were performed with COOT^34^. All structural figures were generated with PyMOL (DeLano Scientific LLC). Schematic representation showing the interaction between LC3B and Nix LIR^S34, 35E^ was generated by LIGPLOT^35^.

## Electronic supplementary material

Supplementary Info
