# Supplementary material for: Phosphorylation of the mitochondrial autophagy receptor Nix enhances its interaction with LC3 proteins
Source: Sci Rep. 2017 Apr 25;7:1131. doi: 10.1038/s41598-017-01258-6 (PMC5430633; doi:10.1038/s41598-017-01258-6)
Supplement: Supplementary file 1 — Supplementary Info [file 41598_2017_1258_MOESM1_ESM.pdf]

Supplementary material

Phosphorylation of the mitochondrial autophagy receptor Nix enhances its interaction with LC3 proteins

Vladimir V. Rogov[1,*], Hironori Suzuki[2,3,*], Mija Marinković[4], Verena Lang[5], Ryuichi Kato[3], Masato Kawasaki[3], Maja Buljubašić[4], Matilda Šprung[6], Natalia Rogova[1], Soichi Wakatsuki[7], Anne Hamacher-Brady[5,8], Volker Dötsch[1,¶], , Ivan Dikić[4,9], Nathan R. Brady[8,10] and Ivana Novak[4, ¶]

Supplementary material figure legends

Figure S1. Characterization of the Nix phosphorylation effect on LC3B interaction by ITC and NMR. (a) K_D values calculated from LC3B CSP upon titration with P0 based on previous data (left plot) and current data (right plot). (b) Overlaid sections of [^{15}N,^{1}H]-TROSY HSQC spectra indicating exchange modes of selected LC3B resonances upon titration with different LIR peptides. Rainbow color-code represents molar ratio LC3B:peptide from 0 (red) to 8 (violet).

Figure S2. Detailed structure of Nix LIR and LC3B interaction. (a) The electron density map of Nix LIR of molecule A. Atoms are shown and colored similarly to Figure 3. Electron density maps of 2Fo-Fc at 1 σ are shown in gray wire frame representation. The N-terminus of LIR (PPAGL) could not be assigned due to weak electron density. (b) The interaction of Glu34 in the Nix LIR (magenta) with the Arg10, Arg11, Lys51 of LC3B (green). The distances (Å) between atoms are shown in dotted black lines. Glu34 forms salt bridges with Lys51 and Arg10. However, the distance between the carboxyl Glu34 and Arg11 is longer, requiring a rotamer change in Arg11 to facilitate an interaction.

Figure S3. Lysine residue 49 in LC3B increases interaction and colocalization with Bnip3. (a) Co-immunoprecipitation of EGFP-LC3B wild type and K49A together with pmCherry-Bnip3-S17,24E, pmCherry-Nix-S34,35E or tRFP-p62 was performed 24 hrs post transfection using α-GFP. GFP, RFP and tRFP were detected via Western blotting and representative blot is shown. Statistical significance was tested vs LC3B wt using Students one-tailed paired t-test for four independent experiments; *, $p \leq 0.05$, **, $p \leq 0.01$. (b) Colocalization of EGFP-LC3B wild type and Bnip3. Scale bar 10 μm, scale bar for zoom images 5 μm.

Figure S4. Mitochondrial colocalization of Nix wild type and mutants with LC3B. (a) Colocalization of Nix mutants with mitochondrial marker Tom20. EGFP-Nix wild

type, ΔLIR, S34,35A, S34,34D and S34,35E (green) show colocalization with endogenous Tom20 (red). (b) Colocalization of Nix mutants with LC3B upon treatment with CCCP. EGFP-Nix wild type, ΔLIR, S34,35A, S34,34D and S34,35E (green) and LC3B (red).

Figure S5. (a) LC3B Arginine 10 and Arginine 11 are necessary for interaction with Nix. Immunoprecipitation of of EGFP-LC3B wild type and R10A or R11A together with pmCherry-Nix wild type, S34,35A and S34,35E mutant or tRFP-p62. Immunoprecipitation was performed 24 hrs post transfection using α-GFP. GFP, RFP and tRFP were detected via Western blotting and representative blot is shown. LC3B R10A and R11A do not coimmunoprecipitate with Nix wild type and mutants; similarly, LC3B R10A and R11A weakly coimmunoprecipitates with p62. (b) Colocalization profiles for Figure 4a, 4d and 4e. The area of plot profiles is shown in zoom images (Figure 4a, 4d and 4e) represented by a white line.

Figure S6. Analysis of mitochondrial removal determined by flow cytometry using GFP-Nix, ΔLIR, S34,35A and S34,35E mutants. (a) Example of flow cytometry parameters used for normalization of the experiments (singlets analysis (left), exclusion of dead cells determined by propidium iodide (middle) and fluorescence intensity (right)). (b) Analysis of fold change between CCCP and CCCP/Bafilomycin A treated cells using GFP-Nix, ΔLIR, S34,35A and S34,35E mutants in flow cytometry. (c) Western blot analysis of cell lysates used in flow cytometry experiment. α-GFP, α-TOM20, α-LC3 and α-actin were used on the representative blot shown.

Figure S7. LC3B Lysine 51 is necessary to recognize phosphorylated Bnip3 LIR. Immunoprecipitation of EGFP-LC3B wild type and K51A together with pmCherry-Bnip3 wild type, 2SA (S17,24A) and -2SE (S17,24E) mutant. Immunoprecipitation was performed 24 hrs post transfection using α-GFP. GFP, RFP and tRFP were detected via Western blotting and representative blot is shown. LC3B K51A does not coimmunoprecipitate with Bnip3 wild type and mutants.

Figure S8. Extended ITC study of the interactions between LC3B variants and different LIR-containing peptides. (a) ITC profiles for the titration of wild type LC3B and LC3B mutants (R11A, K49A and K51A) with phosphorylated Nix LIR (P2) peptide. (b) ITC profiles for the titration of the same LC3B variants with p62 LIR peptide. (c) ITC profiles for the titration of wild type LC3B and LC3B K49A with Nix LIR (P0) peptide (left plot) and with B2L13 LIR peptide (right plot). In each plot the top diagrams display the raw measurements and the bottom diagrams show the integrated heat per titration step.

Figure S1.

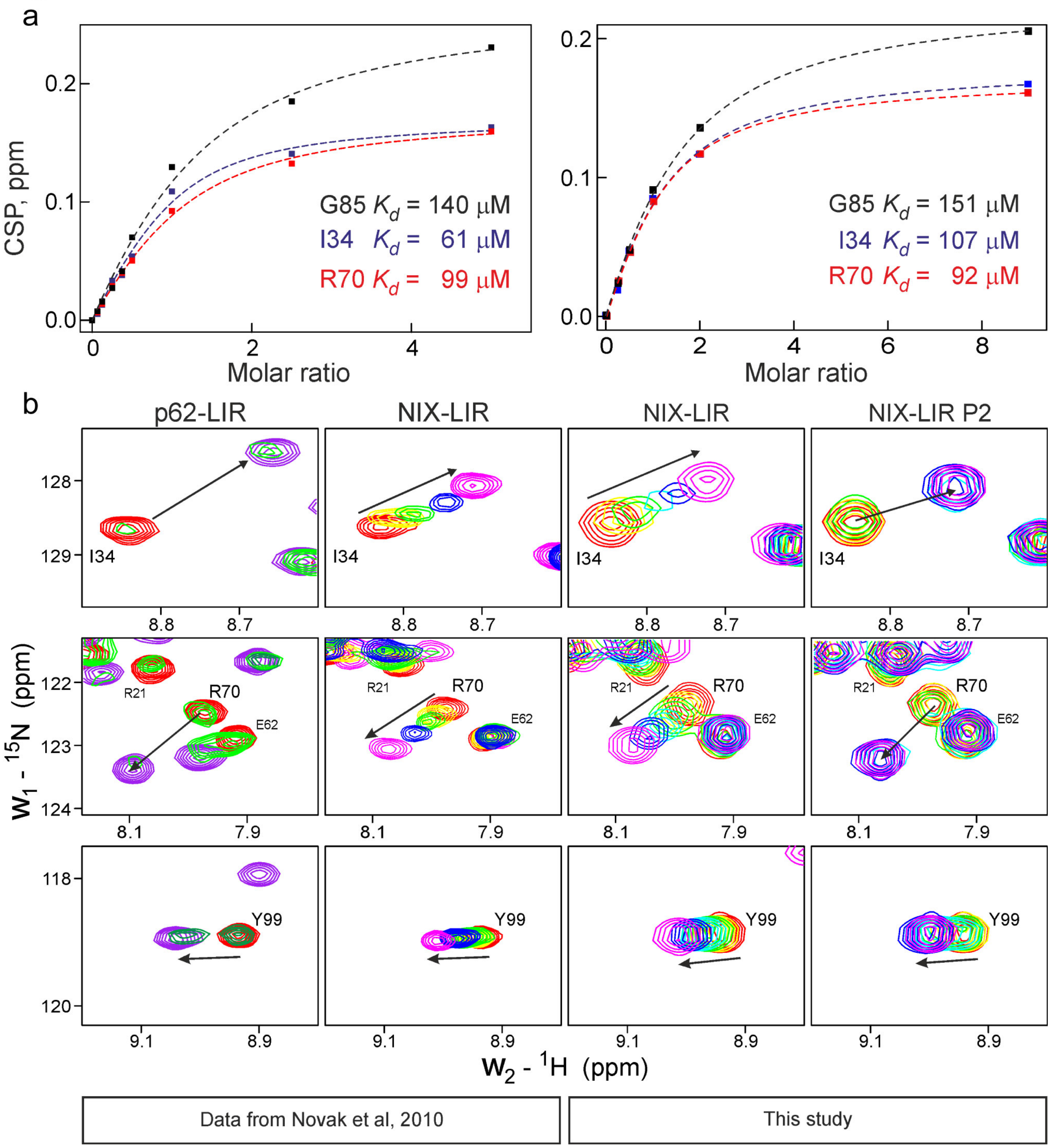

a
CSP, ppm
0.0
0.1
0.2
0
2
4
Molar ratio
G85 K_d = 140 µM
I34 K_d = 61 µM
R70 K_d = 99 µM
0.0
0.1
0.2
0
2
4
6
8
Molar ratio
G85 K_d = 151 µM
I34 K_d = 107 µM
R70 K_d = 92 µM
b
p62-LIR
NIX-LIR
NIX-LIR
NIX-LIR P2
128
129
I34
8.8
8.7
122
123
124
R21
R70
E62
8.1
7.9
118
120
Y99
9.1
8.9
W_1 - ^{15}N (ppm)
W_2 - ^{1}H (ppm)
Data from Novak et al, 2010
This study

Figure S2.

a

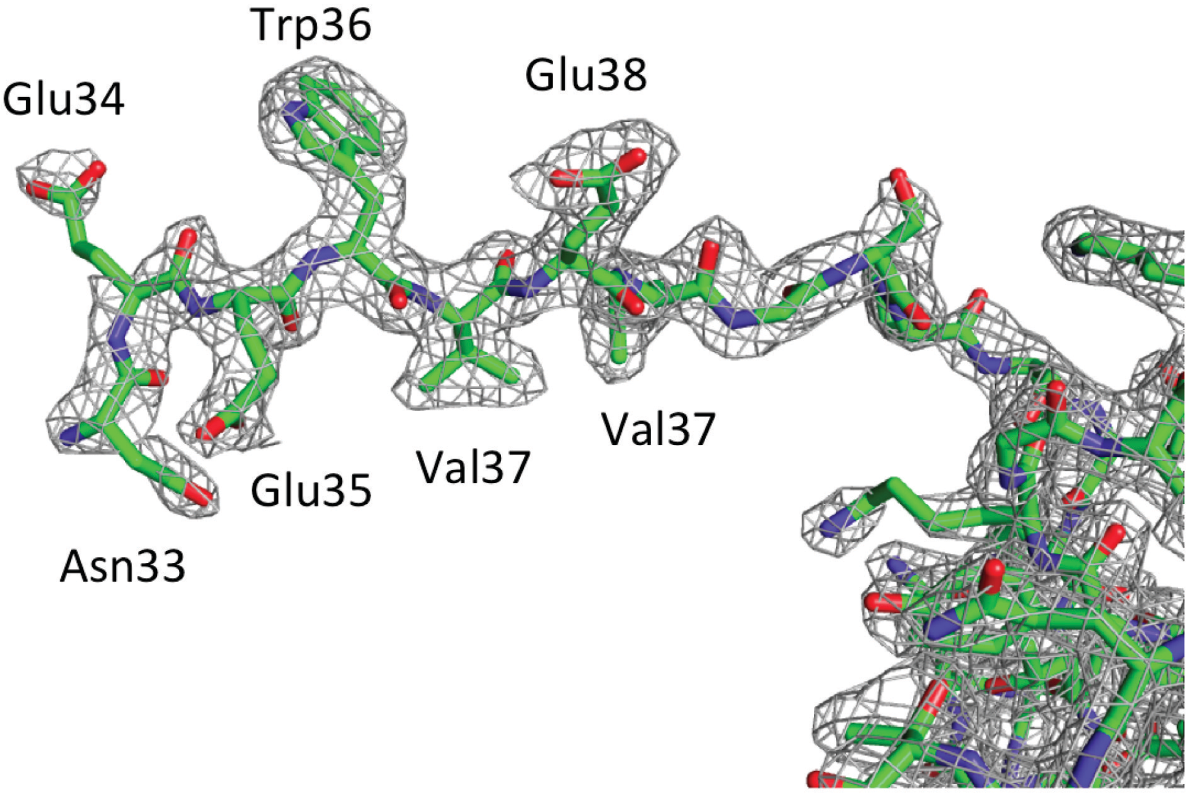

Trp36
Glu34
Glu38
Val37
Glu35
Val37
Asn33

b

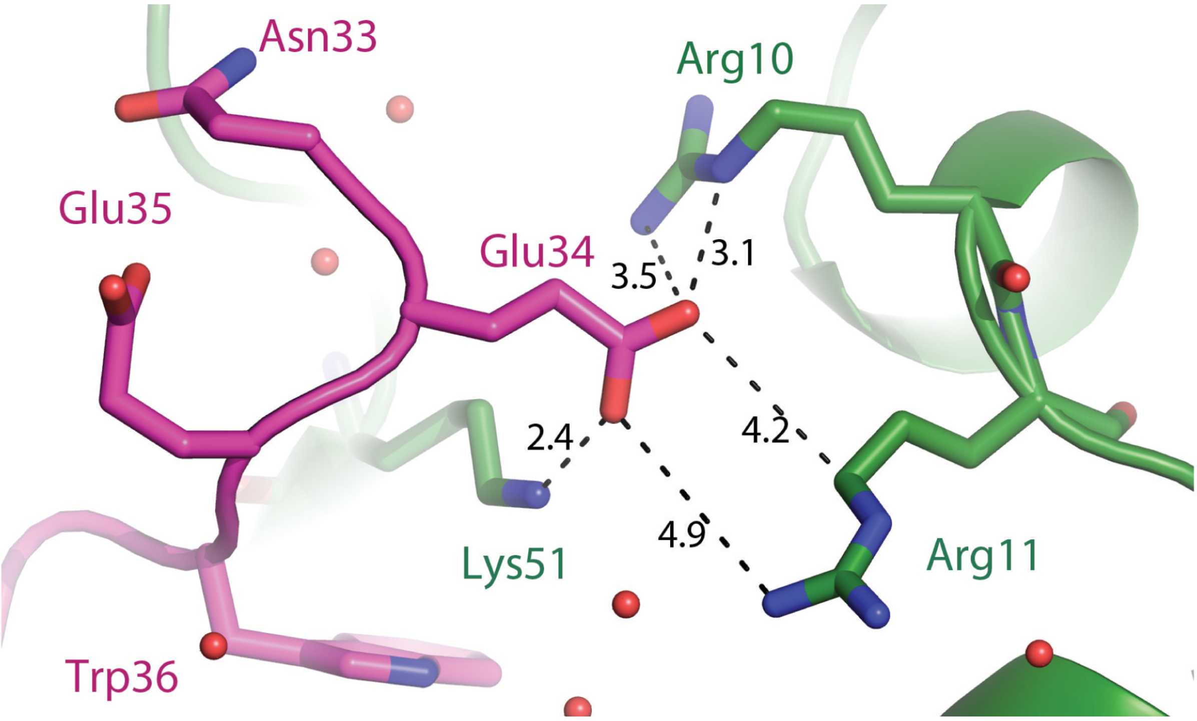

Asn33
Arg10
Glu35
Glu34
3.5
3.1
2.4
4.2
4.9
Lys51
Arg11
Trp36

Figure S3.

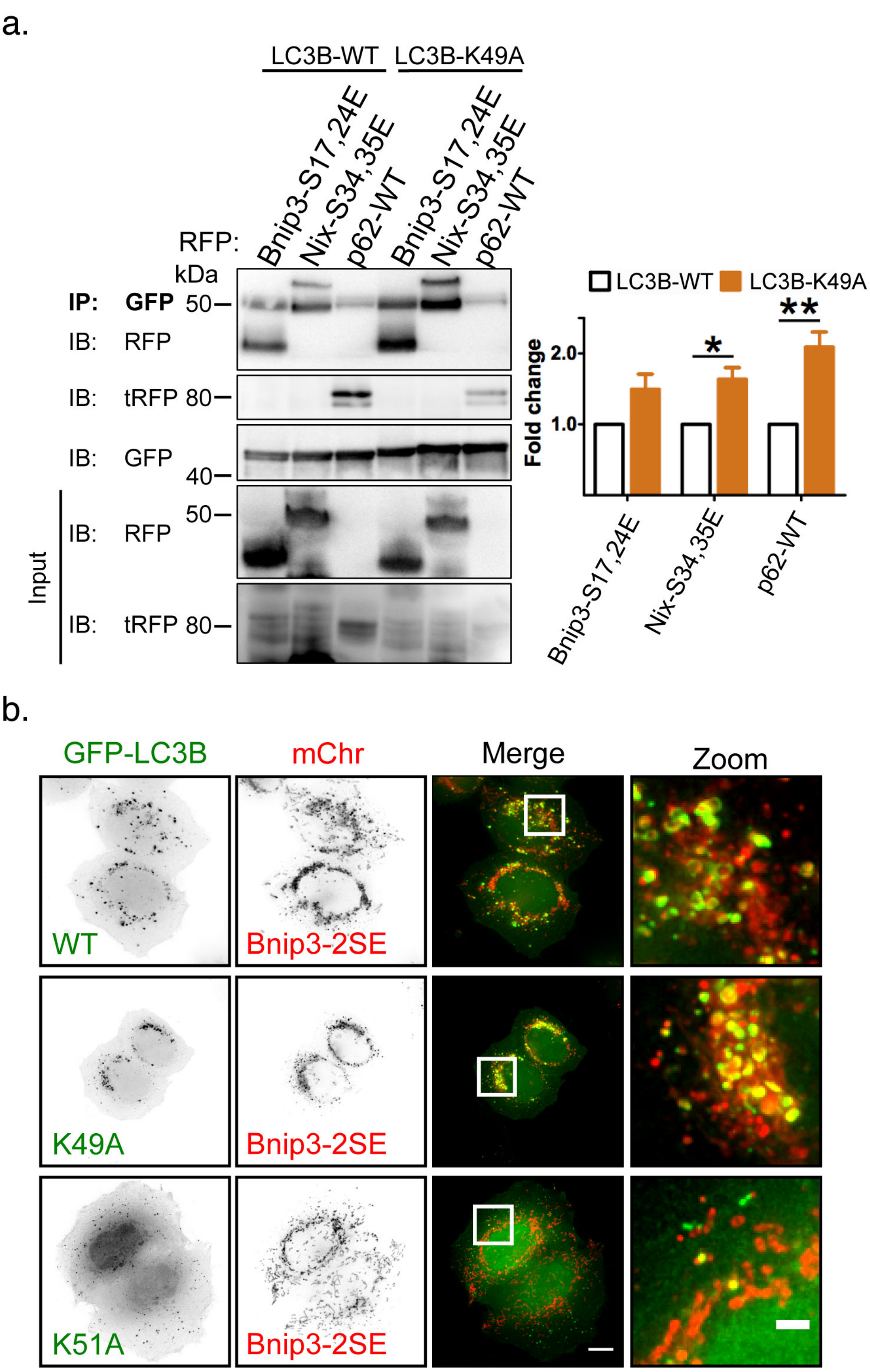
a.
LC3B-WT
LC3B-K49A
RFP:
Bnip3-S17,24E
Nix-S34,35E
p62-WT
Bnip3-S17,24E
Nix-S34,35E
p62-WT
kDa
IP: GFP
IB: RFP
50—
IB: tRFP 80—
IB: GFP
40—
Input
IB: RFP
50—
IB: tRFP 80—
LC3B-WT
LC3B-K49A
Fold change
2.0
1.0
*
**
Bnip3-S17,24E
Nix-S34,35E
p62-WT
b.
GFP-LC3B
mChr
Merge
Zoom
WT
K49A
K51A
Bnip3-2SE
Bnip3-2SE
Bnip3-2SE

Figure S4.

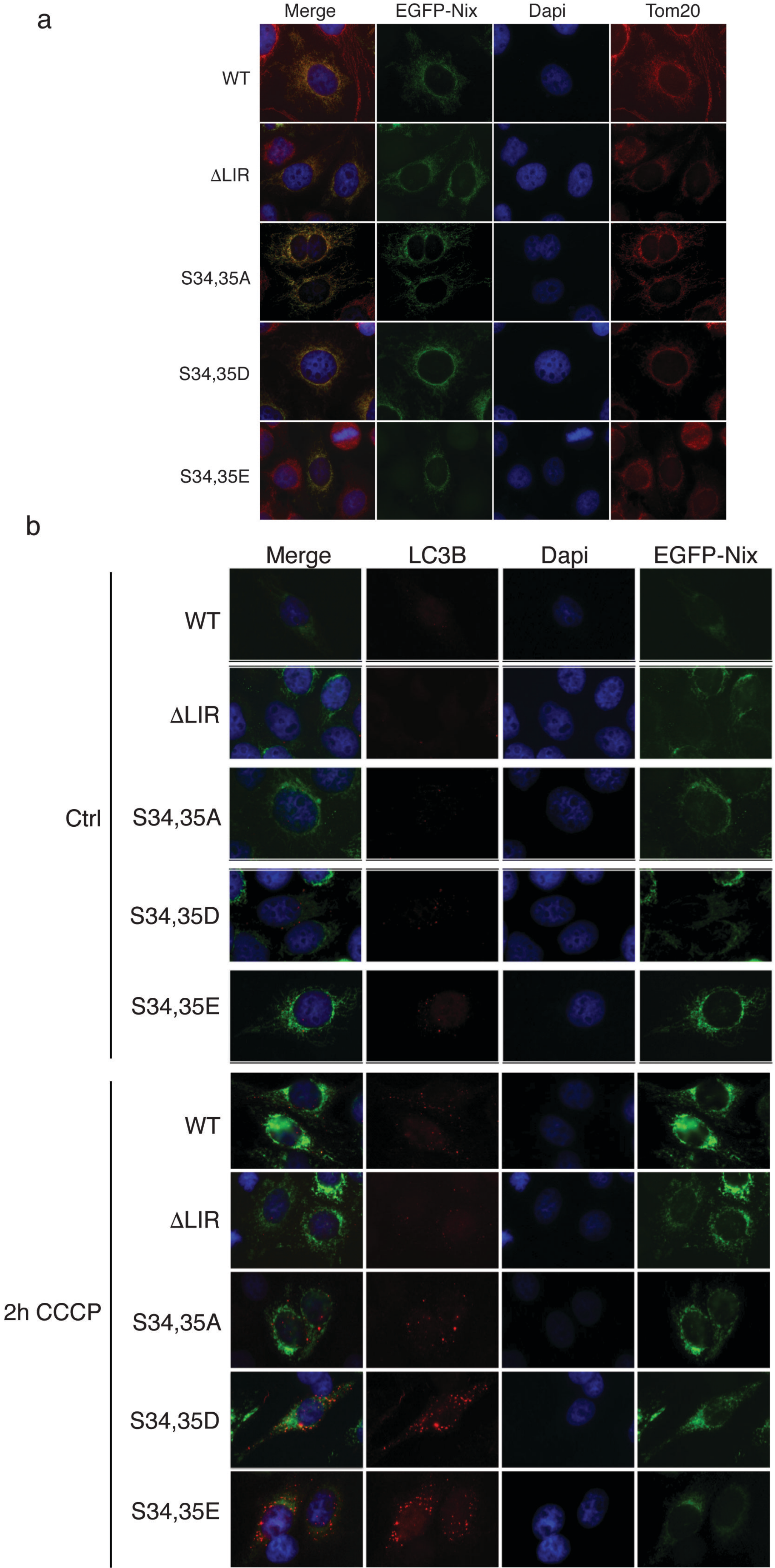
a
Merge
EGFP-Nix
Dapi
Tom20
WT
ΔLIR
S34,35A
S34,35D
S34,35E
b
Merge
LC3B
Dapi
EGFP-Nix
Ctrl
WT
ΔLIR
S34,35A
S34,35D
S34,35E
2h CCCP
WT
ΔLIR
S34,35A
S34,35D
S34,35E

Figure S5.

a

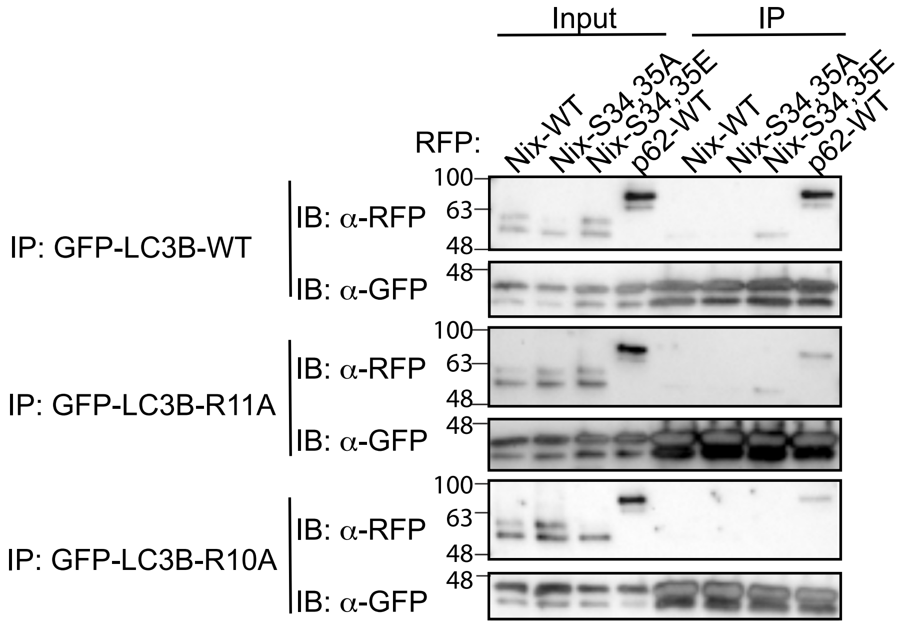
Input
IP
RFP:
Nix-WT
Nix-S34,35A
Nix-S34,35E
p62-WT
Nix-WT
Nix-S34,35A
Nix-S34,35E
p62-WT
IP: GFP-LC3B-WT
IB: α-RFP
IB: α-GFP
IP: GFP-LC3B-R11A
IB: α-RFP
IB: α-GFP
IP: GFP-LC3B-R10A
IB: α-RFP
IB: α-GFP
100
63
48
48
100
63
48
48
100
63
48
48

Figure S5.

b

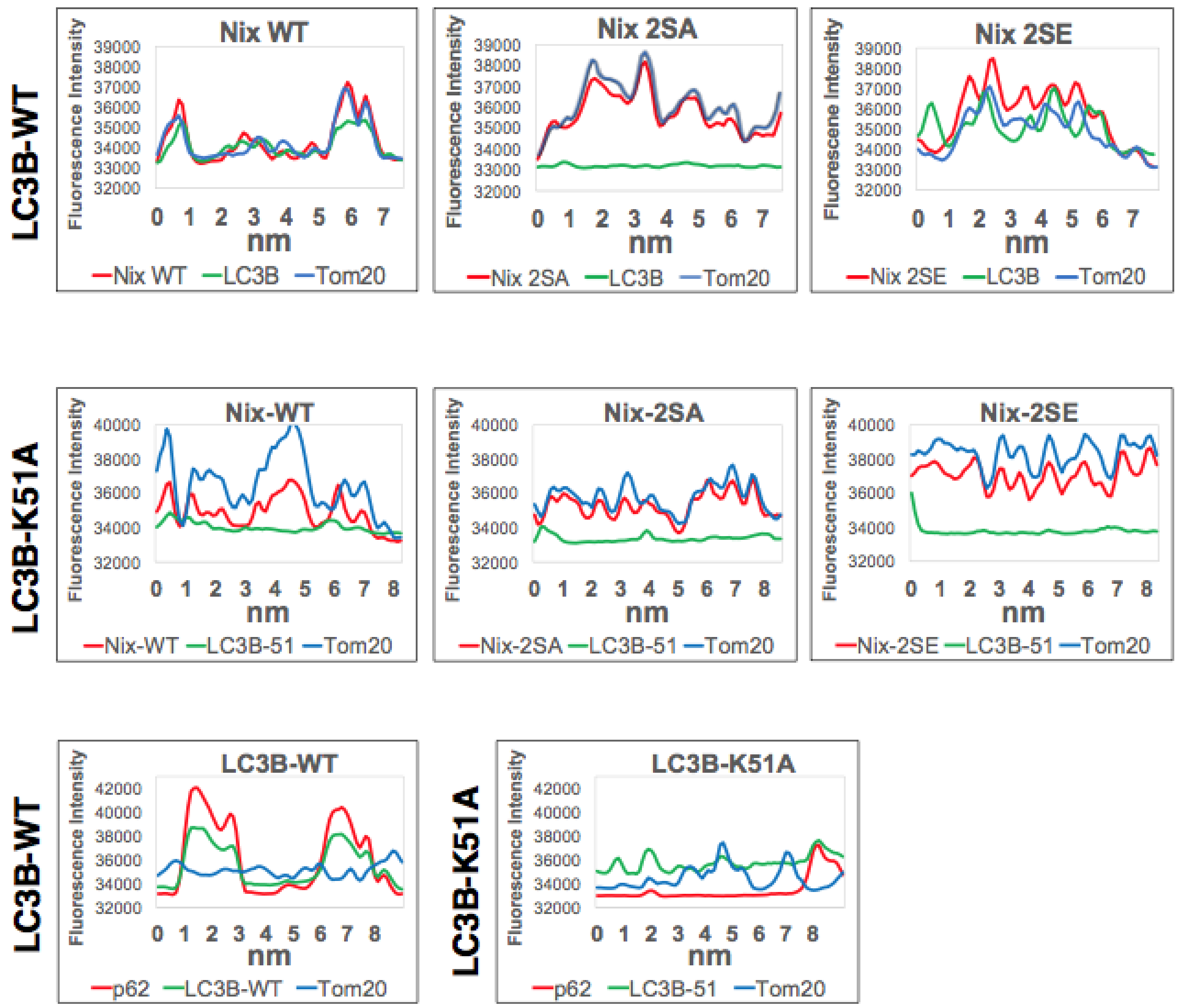
LC3B-WT
Nix WT
Nix 2SA
Nix 2SE
LC3B-K51A
Nix-WT
Nix-2SA
Nix-2SE
LC3B-WT
LC3B-K51A
Fluorescence Intensity
nm
Nix WT LC3B Tom20
Nix 2SA LC3B Tom20
Nix 2SE LC3B Tom20
Nix-WT LC3B-51 Tom20
Nix-2SA LC3B-51 Tom20
Nix-2SE LC3B-51 Tom20
p62 LC3B-WT Tom20
p62 LC3B-51 Tom20

Figure S6.

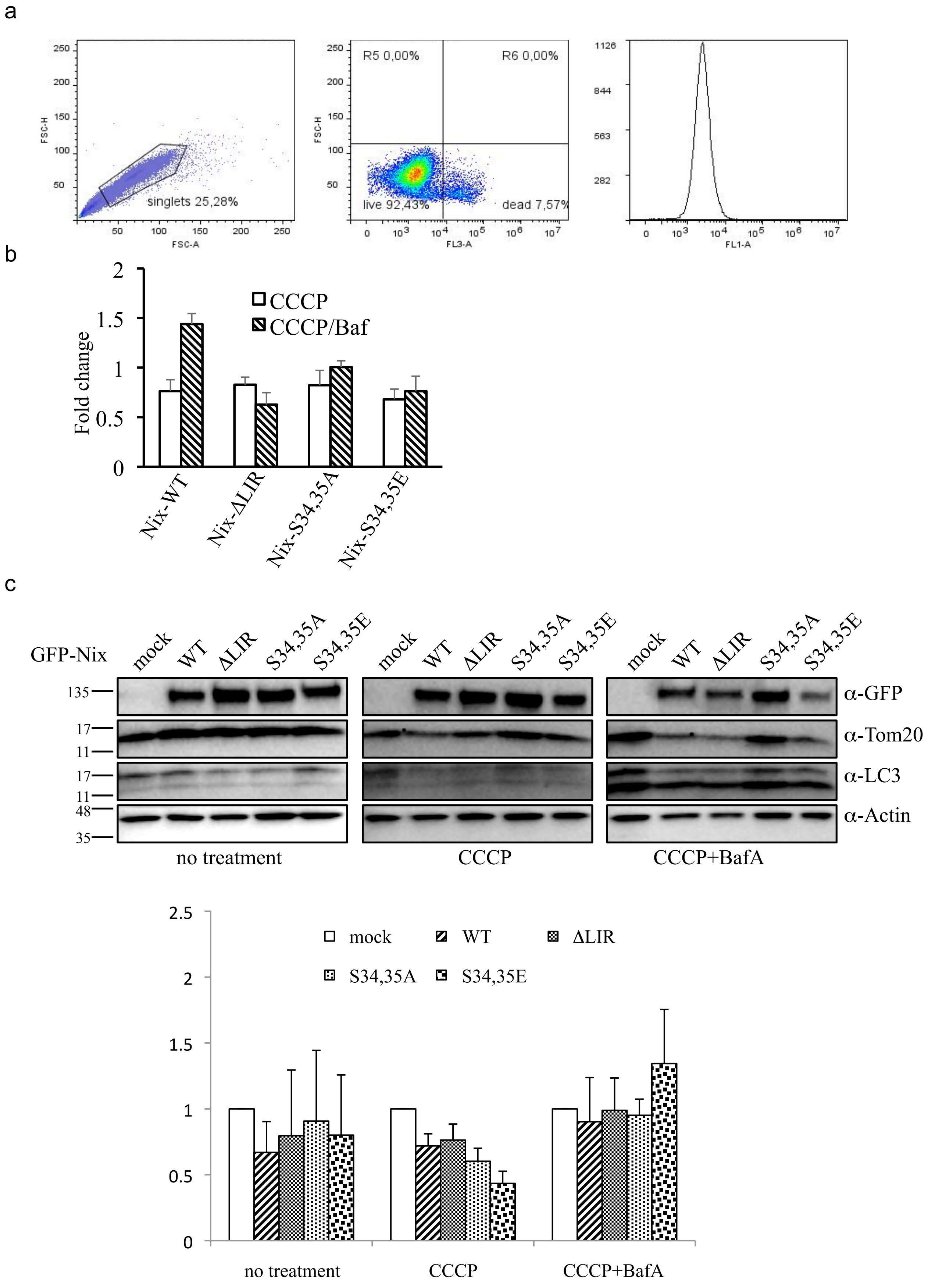

a
singlets 25,28%
FSC-H
FSC-A
R5 0,00%
R6 0,00%
live 92,43%
dead 7,57%
FL3-A
FL1-A
b
Fold change
CCCP
CCCP/Baf
Nix-WT
Nix-ΔLIR
Nix-S34,35A
Nix-S34,35E
c
GFP-Nix
mock
WT
ΔLIR
S34,35A
S34,35E
α-GFP
α-Tom20
α-LC3
α-Actin
no treatment
CCCP
CCCP+BafA

Figure S7.

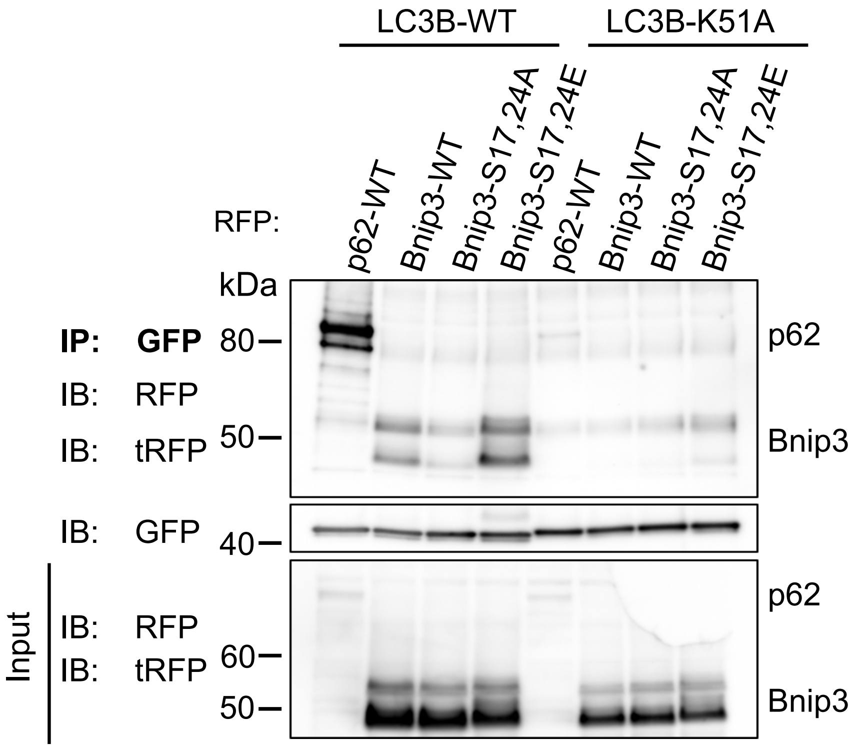
LC3B-WT
LC3B-K51A
RFP:
p62-WT
Bnip3-WT
Bnip3-S17,24A
Bnip3-S17,24E
p62-WT
Bnip3-WT
Bnip3-S17,24A
Bnip3-S17,24E
kDa
IP: GFP
IB: RFP
IB: tRFP
80
50
p62
Bnip3
IB: GFP
40
Input
IB: RFP
IB: tRFP
60
50
p62
Bnip3

Figure S8.

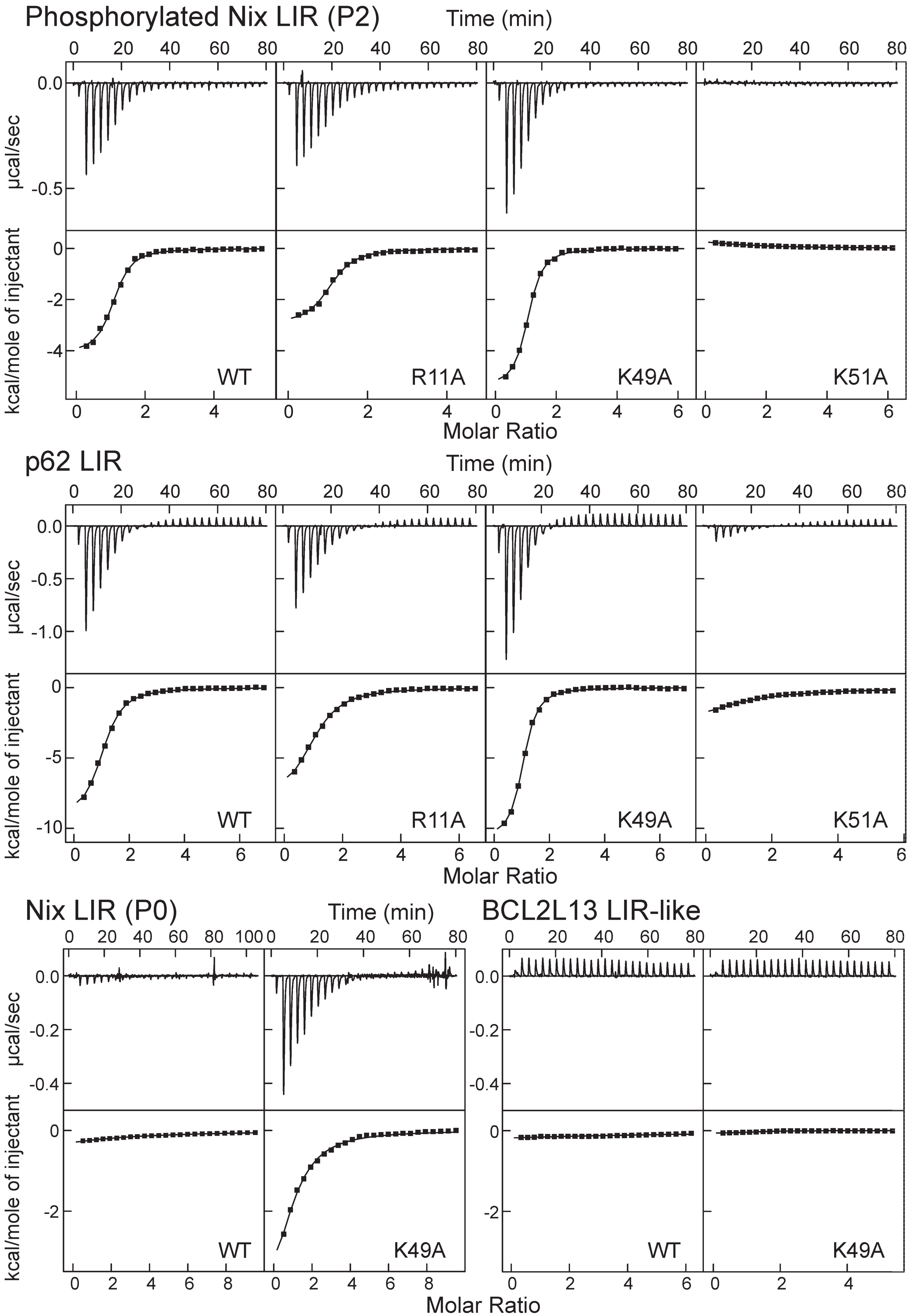

Phosphorylated Nix LIR (P2)
Time (min)
0 20 40 60 80
0.0
-0.5
µcal/sec
0
-2
-4
kcal/mole of injectant
WT
R11A
K49A
K51A
0 2 4 6
Molar Ratio
p62 LIR
Time (min)
0 20 40 60 80
0.0
-0.5
-1.0
µcal/sec
0
-5
-10
kcal/mole of injectant
WT
R11A
K49A
K51A
0 2 4 6
Molar Ratio
Nix LIR (P0)
Time (min)
0 20 40 60 80 100
BCL2L13 LIR-like
0.0
-0.2
-0.4
µcal/sec
0
-2
kcal/mole of injectant
WT
K49A
WT
K49A
0 2 4 6 8
Molar Ratio
